# Amorphous silica nanoparticles cause abnormal cytokinesis and multinucleation through dysfunction of the centralspindlin complex and microfilaments

**DOI:** 10.1186/s12989-023-00544-8

**Published:** 2023-08-22

**Authors:** Liyan Xiao, Jinyan Pang, Hua Qin, Liyang Dou, Man Yang, Ji Wang, Xianqing Zhou, Yang Li, Junchao Duan, Zhiwei Sun

**Affiliations:** 1 Beijing Key Laboratory of Environmental Toxicology, School of Public Health, Capital Medical University, Beijing, 100069 P.R. China; 2 Department of Chemistry, College of Sciences, Northeastern University, 110819 Shenyang, P.R. China; 3 Department of Geriatric Medicine, Medical Health Center, Beijing Friendship Hospital, Capital Medical University, 100050 Beijing, P.R. China

**Keywords:** Amorphous silica nanoparticles, Multinucleation, Cytokinesis, Centralspindlin, Microfilaments, Aurora B

## Abstract

**Background:**

With the large-scale production and application of amorphous silica nanoparticles (aSiNPs), its adverse health effects are more worthy of our attention. Our previous research has demonstrated for the first time that aSiNPs induced cytokinesis failure, which resulted in abnormally high incidences of multinucleation in vitro, but the underlying mechanisms remain unclear. Therefore, the purpose of this study was firstly to explore whether aSiNPs induced multinucleation in vivo, and secondly to investigate the underlying mechanism of how aSiNPs caused abnormal cytokinesis and multinucleation.

**Methods:**

Male ICR mice with intratracheal instillation of aSiNPs were used as an experimental model in vivo. Human hepatic cell line (L-02) was introduced for further mechanism study in vitro.

**Results:**

In vivo, histopathological results showed that the rate of multinucleation was significantly increased in the liver and lung tissue after aSiNPs treatment. In vitro, immunofluorescence results manifested that aSiNPs directly caused microfilaments aggregation. Following mechanism studies indicated that aSiNPs increased ROS levels. The accumulation of ROS further inhibited the PI3k 110β/Aurora B pathway, leading to a decrease in the expression of centralspindlin subunits MKLP1 and CYK4 as well as downstream cytokines regulation related proteins Ect2, Cep55, CHMP2A and RhoA. Meanwhile, the particles caused abnormal co-localization of the key mitotic regulatory kinase Aurora B and the centralspindlin complex by inhibiting the PI3k 110β/Aurora B pathway. PI3K activator IGF increased the phosphorylation level of Aurora B and improved the relative ratio of the centralspindlin cluster. And ROS inhibitors NAC reduced the ratio of multinucleation, alleviated the PI3k 110β/Aurora B pathway inhibition, and then increased the expression of MKLP1, CYK4 and cytokinesis-related proteins, whilst NAC restored the clustering of the centralspindlin.

**Conclusion:**

This study demonstrated that aSiNPs led to multinucleation formation both in vivo and in vitro. ASiNPs exposure caused microfilaments aggregation and inhibited the PI3k 110β/Aurora B pathway through excessive ROS, which then hindered the centralspindlin cluster as well as restrained the expression of centralspindlin subunits and cytokinesis-related proteins, which ultimately resulted in cytokinesis failure and the formation of multinucleation.

**Supplementary Information:**

The online version contains supplementary material available at 10.1186/s12989-023-00544-8.

## Background

Synthetic amorphous silica nanoparticles (aSiNPs) belongs to inorganic engineered materials, ranging in size from 1 to 100 nm [1]. Due to its good biocompatibility, easy surface modification and high synthesis utilization, aSiNPs is widely used in various fields. In the food industry, aSiNPs is applied to processed foods and has been registered by the European Union as a food additive with the code E551 [2, 3]. In cosmetic products, aSiNPs is introduced as a viscosity stabilizer, as well as an opacifying, absorbent, and suspending agent [4]. In biomedicine, it is usually used as a drug additive that can be added to drug formulations to improve the absorption, enhance the retention or slow the release of certain drugs in the body [5]. As aSiNPs is penetrating more and more aspects closely related to human life, its safety and adverse health effect have been of great concern.

More than one decade ago, the Organization for Economic Cooperation and Development (OECD) and National Institute of Environmental Health Sciences (NIEHS) defined aSiNPs as one of the nanomaterials requiring urgent evaluation [6]. Nowadays, a large number of investigations have already confirmed the potential toxicity of aSiNPs [7]. The particles can enter the body through respiration, digestion, skin, injection, etc., and mainly deposit in the liver, spleen, lung, and other organs with relatively abundant capillary networks [8]. The liver was one of the important target organs of aSiNPs. Intravenously administered aSiNPs directly encountered the liver, whereas particles administered through other routes had to first cross biological barriers such as the intestinal epithelium or alveolar epithelium. Once in the systemic circulation, aSiNPs might be extracted from the blood by hepatic Kupffer cells via phagocytosis and internalized by hepatocytes. Our research group and other investigators have demonstrated the hepatotoxicity of aSiNPs in vivo and in vitro [8, 9]. Furthermore, we accidentally found and reported for the first time that aSiNPs could lead to the formation of multinucleated cells in both the human hepatic cell line (L-02) and the human hepatoma cell line (HepG2) [10, 11]. Preliminary observation suggested that abnormal mitosis, especially cytokinesis failure, caused by aSiNPs should be responsible for its multinucleation effect [12].

At the same time, some researchers also reported that other nanomaterials, such as fullerenes, TiO_2_ nanoparticles and gold nanoparticles, could cause cytokinesis arrest as well [11–13]. The formed multinucleated cells, on the one hand, underwent apoptotic or mitotic catastrophe, on the other hand, this might instead result in cell transformation owing to chromosomal instability [13]. Mitosis is a process that involves the cell accurately dividing the replicated chromosomes into two daughter cells. Thus, the smooth completion of mitosis is an essential guarantee for the cells to maintain their own chromosomal stability [14]. Cytokinesis is the final stage of cell mitosis, in which through corresponding plasma membrane remodeling and cytoplasmic division two daughter cells are produced. Cytokinesis arrest leads to the formation of tetraploid cells, if these tetraploid cells do not undergo effective mitotic catastrophe, persistent genome instable aneuploidy will be formed [15]. In the previous study, we observed in the aSiNPs treated group that a small part of L-02 cells could not perform cytokinesis completely, and then combined into abnormal multinucleated cells directly, or there still remained a thin cellular bridge connecting the daughter cells, and these two daughter cells might subsequently combine to one multinucleated cell [12]. However, the mechanism and consequence of abnormal cytokinesis induced by aSiNPs are still unclear.

Cytokinesis is initiated during anaphase, with the chromosomes moving toward opposite ends of the mitotic spindle, while overlapped nokinetochore microtubules form the central spindle between separating chromosomes. This process is followed by the formation of the centralspindlin complex, a key regulator of cytokinesis, which consists of two copies of the kinesin motor protein MKLP1 and the Rho GTPase activating protein CYK4 [16]. At anaphase onset, centralspindlin is strictly localized to the plus ends of antiparallel microtubules, and initiates microtubule bundling as well as central spindle assembly. Then this multifunctional complex further recruits downstream cytokinetic effector proteins to control actinomyosin contractile ring assembly and to promote cleavage furrow ingression and abscission [17]. It was confirmed that only the intact centralspindlin complex could regulate central spindle formation and cytokinesis, neither MKLP1 nor CYK4 alone [17, 18]. Therefore, the precise regulation of centralspindlin complex and the well dynamics of microtubules and microfilaments are two essential aspects to guarantee the cytokinesis progression.

Induction of oxidative stress was one of the recognized toxic modes of aSiNPs. The phenomenon that excessive ROS accelerated telomere shortening and generated chromosome fusions, leading to chromatin bridges and micronucleus formation upon cell division were observed [19]. Our research group had previously confirmed that the excessive production of ROS induced by aSiNPs was related to the formation of multinucleated cells [9]. It was speculated that excessive ROS induced by aSiNPs possibly affected the expression or function of cytokinesis-related proteins, which might be responsible for the abnormal cytokinesis and multinucleated cells. Our previous transcriptomic analyses found that aSiNPs caused a significant down-regulation of the PI3K/Akt signaling pathway. It was demonstrated that excess ROS induced a decrease in phosphorylated PI3K and Akt, ultimately leading to cell cycle arrest, which resulted in the failure of cell division [20]. In addition, shen et al. showed that the downregulation of the PI3K/Akt signaling pathway had an impact on the F-actin cytoskeleton [21]. Therefore, the PI3K/Akt pathway that is closely related to cell division and cytoskeleton regulation could be affected by excessive ROS. Aurora B is a core kinase of the chromosomal passenger complex, which regulates various processes during mitosis [22]. In particular, Aurora B primarily phosphorylates MKLP1, the subunit of centralspindlin, at the highly conserved site S708, which regulates the clustering and localization of centralspindlin [23, 24]. Studies had suggested that PI3K p110β subunit could influence the activity of Aurora B in the metaphase and anaphase, and subsequently regulated mitosis and cytokinesis through this kinase [25]. Taken together, it was hypothesized aSiNPs possibly inhibited PI3K/Aurora B signaling through excessive ROS, which further affected the cytoskeleton, centralspindlin complex, and cytokinesis related proteins. The abnormal regulation of cytokinesis might induce multinucleated cell formation.

The purpose of this study was to explore the underlying mechanisms of how aSiNPs induced abnormal cytokinesis and multinucleation. Animal experiments using ICR mice were first performed to confirm the multinucleation effect of aSiNPs in vivo. For continuous mechanism study, in vitro cultured human hepatic L-02 cells were introduced. The structure and intracellular distribution of microfilaments and microtubules were observed to reflect the potential changes in cytoskeleton function. The protein level of MKLP1, CYK4 and downstream cytokinetic effector proteins, including Ect2, Cep55, CHMP2A and RhoA, was assessed to report the changes of cytokinesis regulatory proteins. In addition, the co-localization of Aurora B and MKLP1, as well as centralspindlin subunits MKLP1 and CYK4 were detected to embody the alteration in centralspindlin complex formation. Furthermore, ROS inhibitor NAC and PI3K activator IGF were used to verify the relationship between aSiNPs causing excessive ROS as well as down regulation of PI3K/Aurora B signaling and the induced abnormal cytokinesis and multinucleation.

## Materials and methods

### Chemicals and antibodies

Acti-stain fluorescent phalloidin (# PHDG1) was purchased from Cytoskeleton. DAPI (D9542) and 2′, 7′-dichlorofluorescein diacetates (DCFH-DA, D6883) were obtained from Sigma-Aldrich. Acetylcysteine (NAC, HY-B0215) was purchased from MedChem Express. Human insulin-like growth factor (IGF, #8917), rabbit anti-PI3K 110α (#4249), rabbit anti-PI3K 110β (#3011), rabbit anti- PI3K p85 (#4292), rabbit anti-phospho-Akt (ser473) (#4060) and rabbit anti-GAPDH (#5174) antibodies were purchased from Cell Signaling Technology. Mouse anti-Aurora B (ab3609), rabbit anti-Aurora B (phospho T232) (ab115793), rabbit anti-MKLP1 (ab9259), goat anti-CYK4 (ab2270), chicken anti-alpha tubulin (ab89984), donkey anti-mouse IgG H&L (Alexa Flour 594) preadsorbed (ab150112), donkey anti-goat IgG H&L (Alexa Flour 594) preadsorbed (ab150136), and donkey anti-rabbit IgG H&L (Alexa Flour 647) preadsorbed (ab150063) antibodies were obtained from Abcam. Rabbit anti-Ect2 (sc-1005), mouse anti-Cep55 (sc-374,051), rabbit anti-CHMP2A (sc-67,227) antibodies and normal rabbit IgG (sc-2027) were purchased from Santa Cruz Biotechnology. Donkey anti-chicken IgG H + L (Alexa Flour 488) conjugated (127,888) antibody was bought from Jackson ImmunoResearch. Goat anti-mouse IgG IRDye 680RD (926-68070) and goat anti-rabbit IgG IRDye 800CW (926-32211) antibodies were obtained from Li-Cor Biosciences.

### Characterization of aSiNPs

The amorphous silica nanoparticles (Nano-Si64 and Nano-Si46) and the red fluorescence-labeled aSiNPs were prepared using original and modified Stöber method as previously described [26]. The morphology and average size of the aSiNPs were observed using transmission electron microscopy (TEM) (JEOL JEM2100, Japan), and the purity was assessed by inductively coupled plasma atomic emission spectrometry (ICP-AES) (Thermo Fisher Scientifi c ARL 3520, Switzerland). A zeta potential granulometer (Malvern Nano-ZS90, UK) was introduced to determine the hydrodynamic size and zeta potential of Nano-Si64 and Nano-Si46, whose final concentrations were 12 mg/ml and 14 mg/ml, respectively, in distilled water and Roswell Park Memorial Institute (RPM I) 1640 culture medium (Gibco, USA).

### In vivo experimental design

Male ICR mice (6-8weeks old) were purchased from the Beijing Vital River Laboratory Animal Technology (Beijing, China). The mice were fed in a specific pathogen-free environment and had free access to sterilized food and water. The room was maintained at 20 ± 2 °C and 60 ± 10% relative humidity with a 12 h light-dark cycle. Before treatment, the mice were not fed overnight. All animal care and experimentations were approved by the Animal Ethics Committee at Capital Medical University (approval number AEEI-2019-003).

After being adapted for 7 days, the male ICR mice were randomly divided into two groups, Nano-Si64 treated group (20 mg/kg⋅bw, n = 10) and control group (same volume of saline, n = 10). ASiNPs was administered via intratracheal instillation under anesthesia (5% chloralhydrate, 0.1ml/10 g BW), once 5-day for 30 days. At the end of the experiment, all animals were sacrificed, and the liver and lung tissues were collected for subsequent study.

### Histopathological analysis

The tissues were processed for histopathological evaluation using standard laboratory procedures. Briefly, the liver and lung were removed and fixed in 10% formalin, embedded in paraffin, sectioned, and stained with hematoxylin and eosin (HE) for histological examination. The slides were light microscopically (Olympus X71-F22PH, Japan) examined.

### Cell culture and exposure to aSiNPs

The human hepatic cell line, L-02, was purchased from the Cell Resource Center, Shanghai Institutes for Biological Sciences (SIBS, China). The cells were maintained in RPMI 1640 culture medium (Gibco, USA) supplemented with 10% fetal bovine serum (Gibco, USA), 100 U/ml penicillin, and 100 µg/ml streptomycin, and were cultured at 37 °C in 5% CO_2_ humidified environment. The cells used in this study were in early passages (10–30).

For experiments, the cells were seeded in Corning® cell culture dishs (D × H 100 mm × 20 mm) at a density of 1 × 10^5^ cells/ml and allowed to attach for 24 h, then treated with aSiNPs suspended in the 1640 culture medium of certain concentrations (10, 20 and 50 µg/ml) for another 24 h. Stock suspension of aSiNPs was dispersed by sonicator (160 W, 20 kHz, 5 min) (Bioruptor UDC-200, Belgium), and diluted to various concentrations, then added to L-02 cells immediately. The cells maintained in 1640 culture medium without aSiNPs were used as the control group. Each group had five replicate wells.

### Multinucleation analysis

The cellular multinucleation was observed by Giemsa staining. This complex dye mainly stains chromatin in the nucleus blue and components in the cytoplasm pink or red. After being treated, L-02 cells were washed twice, and then stained with a Giemsa staining kit (Maxim, China) according to the manufacturer’s instructions. The cellular morphological changes were observed under an optical microscope (Olympus IX81, Japan). The rate of multinucleated cells was determined using images of Giemsa staining. Fields were selected at random, numbers of multinucleated cells out of 2000 cells were counted manually by two independent observers, and the rate of multinucleated cells was then calculated.

### Immunofluorescence analysis

The L-02 cells were seeded in confocal dished and allowed to attach for 24 h, then were treated with 20 µg/ml aSiNPs for another 24 h. After that, the cells were fixed with 4% paraformaldehyde, permeabilized with 0.3% Triton X-100 in PBS for 15 min, and blocked with 5% BSA for 1 h. Next, the cells were successively incubated with primary antibody at 4 °C overnight, fluorescent secondary antibody at room temperature for 1 h, and DAPI at room temperature for 15 min. Finally, the morphological examination was performed using Laser Scanning Confocal Microscope (LSCM) (Leica TCS SP8, Germany).

For cytoskeleton observation, the microfilaments were stained with fluorescent phalloidin (Alexa Flour 488), the microtubules were stained with chicken anti-alpha tubulin primary antibody and donkey anti-chicken fluorescent secondary antibody (Alexa Flour 488), the chromosomes or nuclei were stained by DAPI. For the co-localization of Aurora B and MKLP1, the Aurora B was stained with mouse anti-Aurora B primary antibody and donkey anti-mouse fluorescent secondary antibody (Alexa Flour 594), the MKLP1 was stained by rabbit anti-MKLP1 primary antibody and donkey anti-rabbit fluorescent secondary antibody (Alexa Flour 647), the microtubules were stain with chicken anti-alpha tubulin primary antibody and donkey anti-chicken fluorescent secondary antibody (Alexa Flour 488), the chromosomes or nuclei were stained by DAPI. For the co-localization of MKLP1 and CYK4, the MKLP1 was stained by rabbit anti-MKLP1 primary antibody and donkey anti-rabbit fluorescent secondary antibody (Alexa Flour 647), the CYK4 was stained by using goat anti-CYK4 primary antibody and donkey anti-goat fluorescent secondary antibody (Alexa Flour 594), the microtubules were stain with chicken anti-alpha tubulin primary antibody and donkey anti-chicken fluorescent secondary antibody (Alexa Flour 488), the chromosomes or nuclei were stained by DAPI.

### Western blot analysis

The total cellular protein lysate was prepared by lysing cells in RIPA lysis buffer with protease inhibitor and phosphatase inhibitor (Beyotime, China). Total cellular protein content was determined by a BCA protein assay kit (DingGuoBioTECH, China). Equal amounts of lysate proteins were separated with SDS-PAGE (12% separation gels) and transferred to polyvinylidene fluoride (PVDF) membranes (Millipore, USA). After blocking with 5% skim milk in tris-buffered saline (TBS) containing 0.05% Tween-20 (TBST) for 1 h at room temperature, the membranes were incubated with different primary antibodies overnight at 4 °C. The next day, the membranes were washed with TBST three times, and incubated with corresponding IRDye-labeled secondary antibody at room temperature for 1 h. Finally, the protein bands were scanned using the Li-COR Odyssey system (LI-COR Biosciences, USA). Grayscale analysis of the western blots was performed using Odyssey Infrared Imaging software. At least 3 independent experiments were performed and representative results were shown.

### G-LISA rho activation analysis

The intracellular Rho activity was detected by using a commercial G-LISA Rho activation assay biochem kit (#BK121, Cytoskeleton, USA) according to the manufacturer’s instructions. The activity of Rho was reflected by fluorescence intensity which could detect through a microplate luminescence reader (SYNERGY multi-mode reader, BioTek Instruments, USA).

### Co-immunoprecipitation analysis

The total cellular protein lysates were prepared by lysing cells in Triton X-100 lysis buffer (150 mM NaCl, 1% Triton X-100, 50 mM Tris HCl pH 8.0) with protease inhibitor and phosphatase inhibitor (Beyotime, China). The MKLP1 proteins were immunoprecipitated by mixing the lysates with anti-MKLP1 IgG antibody and µMACS Protein G MicroBeads (#130-071-101, Miltenyi Biotec, Germany). The magnetic microbead-antibody-MKLP1 complexes were collected using µ columns (#130-042-701, Miltenyi Biotec, Germany) and µMACS separation unit (#130-042-602, Miltenyi Biotec, Germany). Next, the columns were rinsed with lysis buffer and low-salt wash buffer, then applied pre-heated 1×SDS gel loading buffer onto the column matrix for elution. This eluted immunoprecipitate was subjected to western blot analysis using anti-MKLP1 and anti-CYK4 antibodies. The relative ratio of CYK4 bound to MKLP1 was calculated to reflect the formation of centralspindlin cluster.

### Intracellular ROS analysis

The intracellular ROS level was detected by DCFH-DA, a non-polar compound, which could enter cells and be hydrolyzed into the polar form DCFH. The intracellular DCFH was an oxidation-sensitive fluorescent probe, which could be oxidized by ROS to produce fluorescent DCF. Thus, the fluorescence intensity of DCF was positively correlated with the intracellular ROS quantity. After being treated with different concentrations of aSiNPs or aSiNPs plus NAC for 24 h, the cells were washed with PBS and incubated with 10 µM DCFH-DA (Sigma, USA) at 37 °C for 20 min. The fluorescence intensities were measured by FCM (Becton Dickinson, USA), with an excitation wavelength of 488 nm and an emission wavelength of 525 nm.

### Statistical analysis

Data of multinucleated cells were expressed as frequency and analyzed by chi-square test. Other data were expressed as mean ± S.D. and significance was determined by using one-way analysis of variance (ANOVA) followed by the least significant difference (LSD) test to compare the differences between groups. Differences were considered significant at *p* < 0.05.

## Results

### Characterization of aSiNPs

The two sizes of aSiNPs (64 nm and 46 nm), namely Nano-Si64 and Nano-Si46, have been fully characterized in our previous study [27]. Images of TEM displayed the spherical or ellipsoidal shape of the nanoparticles (Supplementary Fig. 1). The purity of both Nano-Si64 and Nano-Si46 detected by ICP-AES, was higher than 99.9%. Their hydrodynamic diameter and zeta potential were measured in distilled water as the stock medium and in RPMI 1640 culture medium as the exposure medium at 0, 3, 6, 12 and 24 h to reflect their dispersion throughout the experiments. At the same time, the hydrodynamic diameter and zeta potential of Nano-Si64 were measured in physiological saline (exposure medium) at 10 min, 1, 6, 12, 24 h to reflect it dispersion throughout the experiments. The hydrodynamic diameter of the Nano-Si64 and Nano-Si46 were approximately 109 nm and 67 nm. It had not changed significantly with time. Zeta potential measurement showed that these particles were highly negatively charged (about − 30 mV) (Supplementary Table 1). The results indicated that the two aSiNPs maintained fairly good monodispersity in both storage medium and experimental medium.

### Multinucleation induced by aSiNPs in vivo and in vitro

**Fig. 1:**
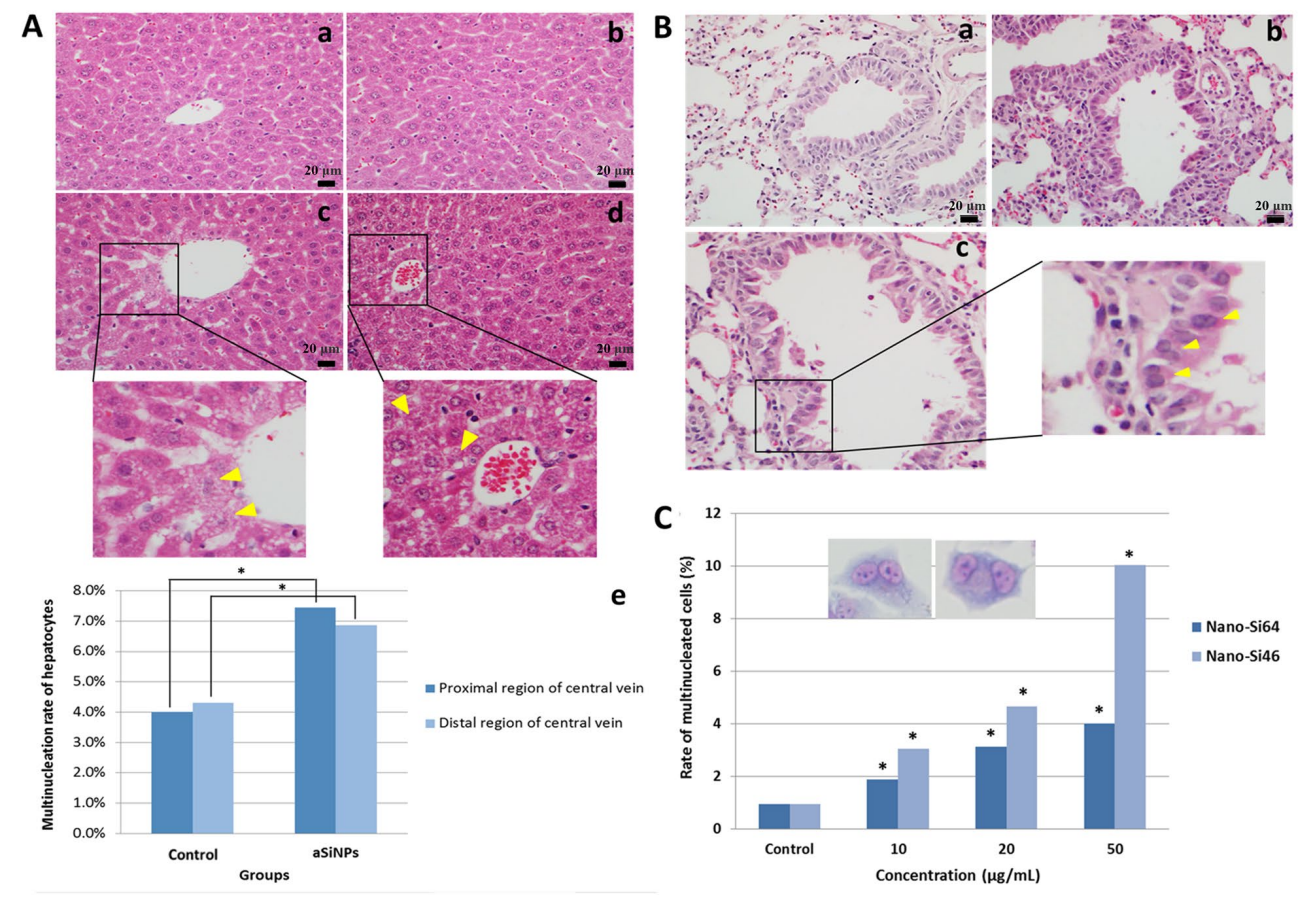
Multinucleation induced by aSiNPs both in vivo and in vitro. Pathological analyses of the liver and lung tissue were performed after the mice were intratracheally instilled with aSiNPs (**A** and **B**). (**A**) Representative images of pathological changes in the liver, (**a**) and (**b**) control group, (**c**) and (**d**) aSiNPs treated group, yellow arrow heads: nuclear fragmentation, vacuolization and necrosis of hepatocytes, (**e**) multinucleation rate of hepatocytes in proximal region and distal region of central vein, and data were expressed as frequency. (**B**) Representative images of pathological changes in the lung tissue, (**a**) control group, (**b**) aSiNPs treated group, (**c**) magnified images of multinucleated bronchial epithelial cells (yellow arrow heads). (**C**) Rate of multinucleated cells induced by aSiNPs in L-02 cells in vitro, and data were expressed as frequency. * *p* < 0.05 compared with control group using chi-square test

After the mice were intratracheally instilled with 64 nm aSiNPs, the pathological analyses of the liver and lung tissue were performed. As shown in Fig. 1A, the structure of the liver lobules was visible, and the hepatocyte cords were arranged radially around the central vein in the control group. While in the aSiNPs treated group, nuclear fragmentation, vacuolization and necrosis of hepatocytes were observed near the central vein. Additionally, the rate of multinucleated hepatocytes increased significantly in the aSiNPs treated group in both the proximal region and distal region of the central vein (Fig. 1Ae). After treated with aSiNPs, the proportion of multinucleated hepatocytes in the proximal region of the central vein was up to 7.5%, which was significantly higher than the proportion of multinucleation in the control group (around 4%). Similarly, there were 6.8% multinucleated cells in the aSiNPs treated group in the distal region of the central vein, where the proportion was approximately 1.6 times higher than that in the control group. Figure 1B displayed the HE staining images of lung tissue, where lymphocyte infiltration, pulmonary interstitial thickening and bronchial epithelial damage were observed in the aSiNPs treated group, and a few multinucleate cells were found in bronchial epithelium (Fig. 1Bc). The above results revealed that repeated intratracheal instillation of aSiNPs not only damaged lung tissue, but also meant the particles could penetrate the liver through blood circulation and cause liver injury as well as multinucleation of hepatocytes.

The multinucleation effect of aSiNPs was detected in vitro as well. As shown in Fig. 1C, both Nano-Si64 and Nano-Si46 caused the formation of multinucleated cells in the L-02 cell line, and the rate of multinucleated cells increased along the particle dose. In 10 µg/ml and 20 µg/ml aSiNPs treated group, the rate gap of multinucleated cells between two particles was narrow. Moreover, in the highest concentration (50 µg/ml), the multinucleation effect of smaller aSiNPs (Nano-Si46) was significantly stronger than that of Nano-Si64, the rate of multinucleated cells in the Nano-Si46 treatment group was approximately 10%, which was obviously higher than the rate of multinucleation in the Nano-Si64 group (around 4%). Thus, 46 nm aSiNPs was used for subsequent in vitro studies to explore the underlying mechanisms of how aSiNPs induced an increased multinucleation rate in hepatocytes.

### Microfilaments agglomeration induced by aSiNPs

In the previous study, we have already confirmed that abnormal cytokinesis induced by aSiNPs should be responsible for the formation of multinucleated cells. To further investigate the potential effect of aSiNPs on the cytoskeleton and cytokinesis, we synthesized SiO_2_ nanoparticles with red fluorescence encapsulated in the core.

**Fig. 2:**
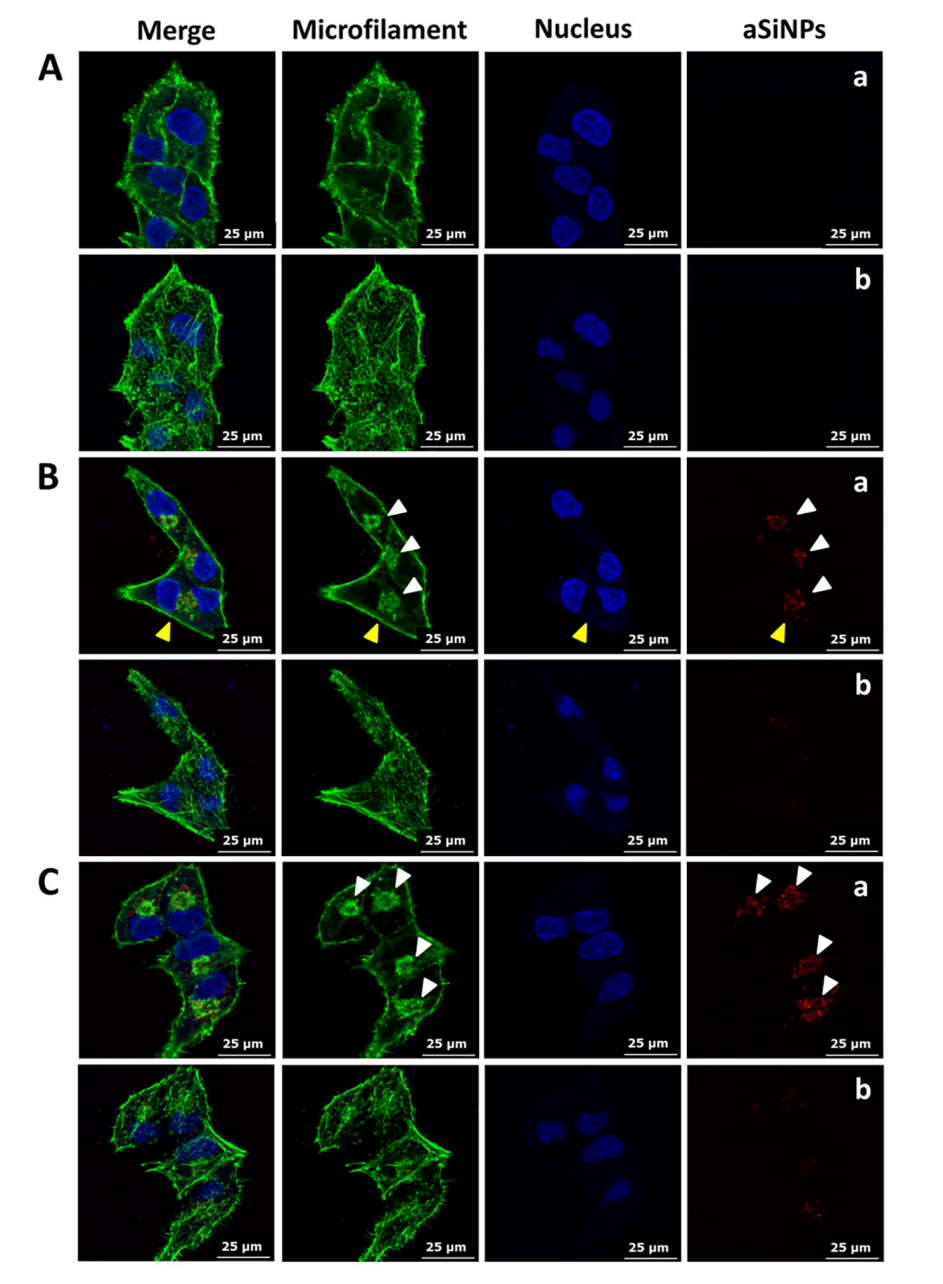
Intracellular distribution of aSiNPs and their effect on the structure and distribution of microfilaments (green: microfilament, blue: nucleus, red: fluorescent silica nanoparticles). (**A**) Immunofluorescence images of L-02 cells in control group, (**a**) nucleus transverse section, (**b**) cell upper surface transverse section. (**B**) and (**C**) Immunofluorescence images of L-02 cells in aSiNPs treated group (20 µg/ml), (**a**) nucleus transverse section, (**b**) cell upper surface transverse section. Yellow arrow head: a multinucleated cell induced by aSiNPs. White arrow heads: agglomerated microfilaments and the co-localized aSiNPs.

The effects of aSiNPs on the structure and cellular distribution of microfilaments were observed by LSCM. As manifested in Fig. 2A, in the control group, microfilaments as a kind of tiny filament were arranged in a network under the cell membrane. In the aSiNPs treated group, L-02 cells could take in the red fluorescence-labeled aSiNPs after 24 h treatment, while their morphology was not affected by 20 µg/ml aSiNPs (Fig. 2B C). To further reveal the cellular localization of aSiNPs, photos from both the nucleus and upper cell surface transverse section were taken. Most of the particles were found near the center of the cells (Fig. 2Ba and 2Ca), and only a small amount of aSiNPs adhered to or were located near the cell surface (Fig. 2BCb). Beyond that, microfilaments agglomeration and an obvious co-localization between aSiNPs and the agglomerated microfilaments were identified (Fig. 2Ba and 2Ca), which indicated that the particles could directly interact with the microfilaments in the cytoplasm and resulted in significant changes in the structure and intracellular distribution of microfilaments. In general, microfilaments can be bundled or scattered in the cytoplasm, which have relationships with cell morphology maintenance, cell and organelle movement, cell division and so on. Therefore, microfilaments aggregation resulted from aSiNPs was likely to cause a series of adverse consequences, including abnormal mitosis.

### Abnormal microtubules distribution and cytokinesis induced by aSiNPs

**Fig. 3:**
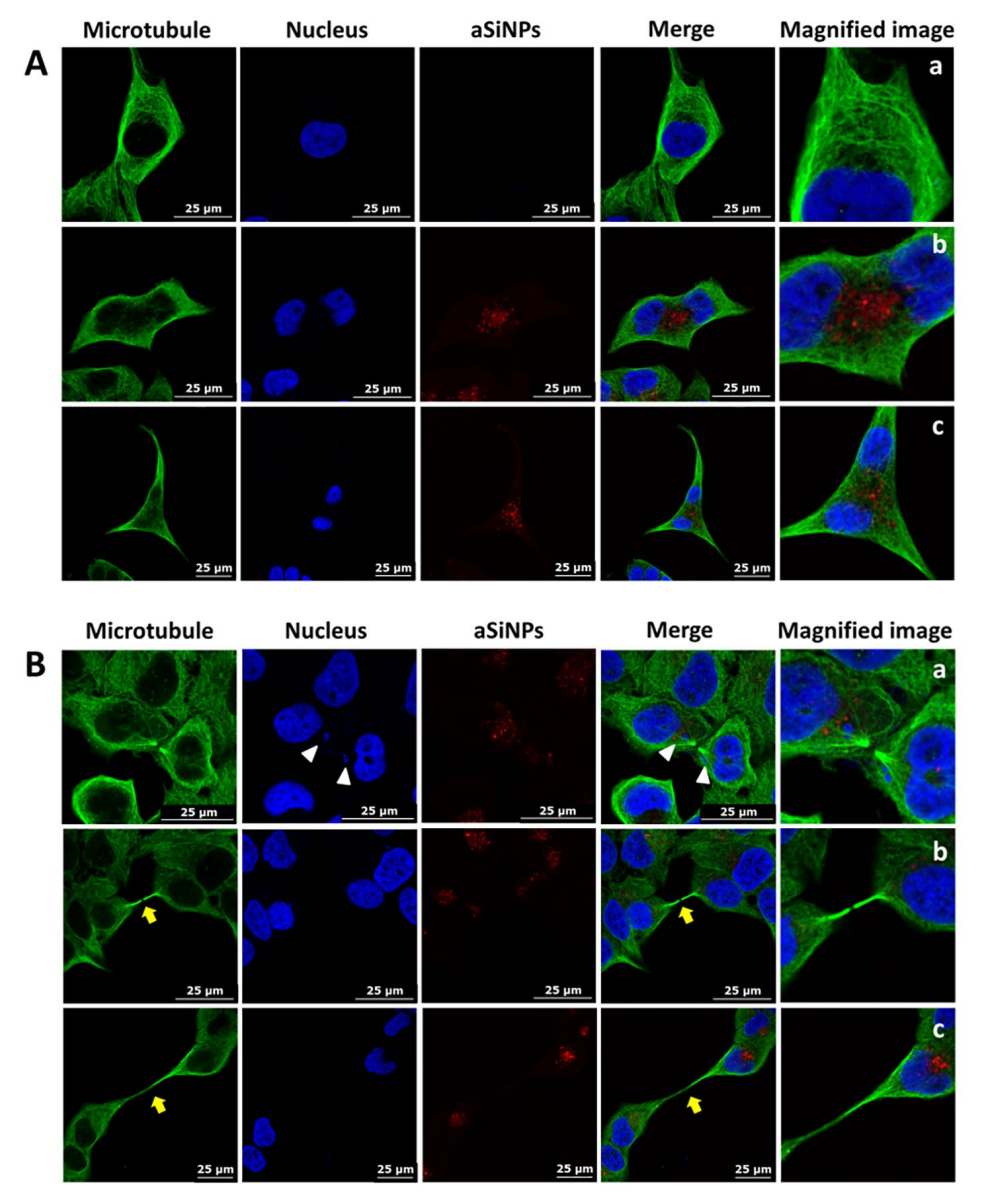
Effects of aSiNPs on microtubules distribution and cytokinesis (green: microtubule, blue: nucleus, red: fluorescent silica nanoparticles). (**A**) Abnormal distribution of microtubules induced by aSiNPs (20 µg/ml), (**a**) normal cells in control group, (**b**) and (**c**) multinucleated cells in aSiNPs treated group. Due to the occupation of agglomerated microfilaments, microtubules were distributed in the cytoplasmic in a network form except the region of agglomerated microfilaments. (**B**) Abnormal cytokinesis induced by aSiNPs (20 µg/ml), (**a**) chromosome fragments (white arrow heads) in daughter cells, (**b**) and (**c**) a thin and long cellular bridge (yellow arrows) connecting the daughter cells

The influence of aSiNPs on microtubules was shown in Fig. 3A. In the control group, the microtubules mainly distributed around the nucleus, forming a network and extending radially to the periphery (Fig. 3Aa). In the aSiNPs treated group, the morphology and structure of microtubules were not changed significantly, but due to the occupation of agglomerated microfilaments, the distribution of microtubules in the perinuclear cytoplasmic region was notably affected (Fig. 3Ab and 3Ac).

Abnormal cytokinesis induced by aSiNPs was displayed in Fig. 3B. At the terminal stage of mitosis, the formed constriction could not separate the two daughter cells effectively, instead leaving a thin and long intercellular bridge between them (Fig. 3B yellow arrows). These incomplete cytokinesis cells might recombine into one multinucleated cell and double the number of chromosomes, seeming like the binucleated cells shown in Fig. 3Ab and 3Ac. In addition, owing to the disappearance of the nuclear membrane during mitosis, aSiNPs entering the cells could directly interact with the chromosomes, causing chromosome breakage, and micronuclei formation outside the main nucleus in daughter cells (Fig. 3B white arrow heads). Thus, numerical aberration of chromosomes followed by cytokinesis failure, as well as chromosome damage caused by aSiNPs would result in a further increase in chromosomal instability (CIN) of L-02 cells.

### Down-regulation of the PI3K 110β/Aurora B pathway and cytokinesis regulatory proteins induced by aSiNPs

**Fig. 4:**
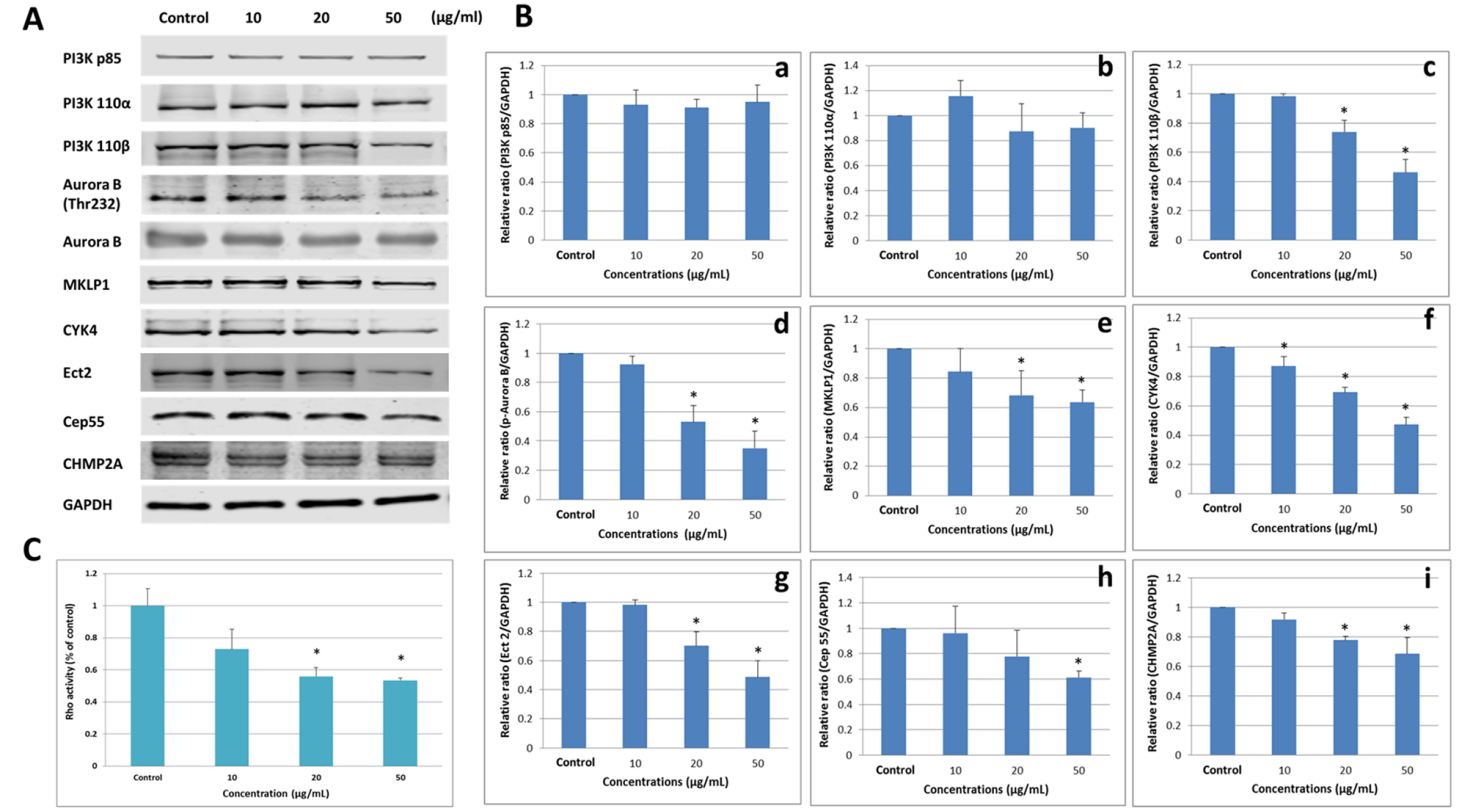
The expression of proteins related to PI3K/Aurora B pathway and cytokinesis regulation in L-02 cells after 24 h exposure to aSiNPs with different concentrations. (**A**) Results of western blot analysis. GAPDH was used as an internal control to monitor for equal loading. (**B**) Relative densitometric analysis of the protein bands was performed and presented. (**a**) PI3K p85, (**b**) PI3K 110α, (**c**) PI3K 110β, (**d**) p-Aurora B, (**e**) MKLP1, (**f**) CYK4, (**g**) Ect2, (**h**) Cep55, (**i**) CHMP2A. (**C**) Rho activity detected by G-LISA biochem kit. ASiNPs induced the inhibition of PI3K 110β/Aurora B pathway and cytokinesis related regulation proteins in a dose-dependent way. Data were expressed as means ± SD from three independent experiments * *p* < 0.05 compared with control group using one-way ANOVA.

To better understand the mechanism of cytokinesis failure caused by aSiNPs, the protein expression of mitotic regulatory pathway PI3K/Aurora B signaling and cytokinesis regulators in L-02 cells was examined. Firstly, the cells were treated with different concentrations of aSiNPs (10, 20 and 50 µg/ml) for 24 h, and the protein expression levels of the catalytic subunit p85 and the regulatory subunits p110α and p110β of PI3K were detected. As shown in Fig. 4, the expression of PI3K 110β was down regulated by 20 and 50 µg/ml aSiNPs, but PI3K p85 and PI3K 110α were not affected by aSiNPs. Next, the phosphorylation level of Aurora B was also detected, which is a key mitotic kinase and could be regulated by PI3K 110β. The expression of phosphorylated Aurora B showed a downward trend after aSiNPs treatment as well, and aSiNPs apparently decreased the phosphorylation level of Aurora B at 20 and 50 µg/ml. Thus, the results possibly indicated that aSiNPs inhibited the regulatory activity of Aurora B in mitosis through the p110β subunit of PI3K.

As known, the centralspindlin complex plays a pivotal role in cytokinesis regulation, which is comprised of two subunits, MKLP1 and CYK-4. It was reported that Aurora B regulated the centralspindlin complex by phosphorylating the S708 site of MKLP1, thereby controlling the formation and bundling of the central spindle during anaphase and telophase of mitosis [23, 24]. Therefore, the protein contents of MKLP1, CYK4, and the other four key proteins that manipulated cell division downstream of centralspindlin were detected. The protein level of MKLP1, CYK4, Ect2, Cep55, and CHMP2A was assessed by western blot (Fig. 4A and B), and the activity of the Rho enzyme was assessed by a G-LISA kit (Fig. 4C).

The results in Fig. 4 suggested that compared with the control, aSiNPs evidently decreased the expression of MKLP1 and CYK4. Cep55 and CHMP2A, downstream of MKLP1, are involved in central spindle fascicle formation, and the protein expression of which also decreased in a dose dependent way. Moreover, a similar trend was found both in the Ect2 expression and Rho activity. These two proteins, downstream of CYK4, are concerned to play key roles in the regulation of contractile ring and daughter cell shedding. Thus, the above observations demonstrated that aSiNPs could inhibit PI3K/Aurora B signaling pathway and reduce the content or activity of cytokinesis regulating proteins in L-02 cells, therefore causing the disorder or failure of cytokinesis.

### Abnormal co-localization of Aurora B and centralspindlin complex induced by aSiNPs

**Fig. 5:**
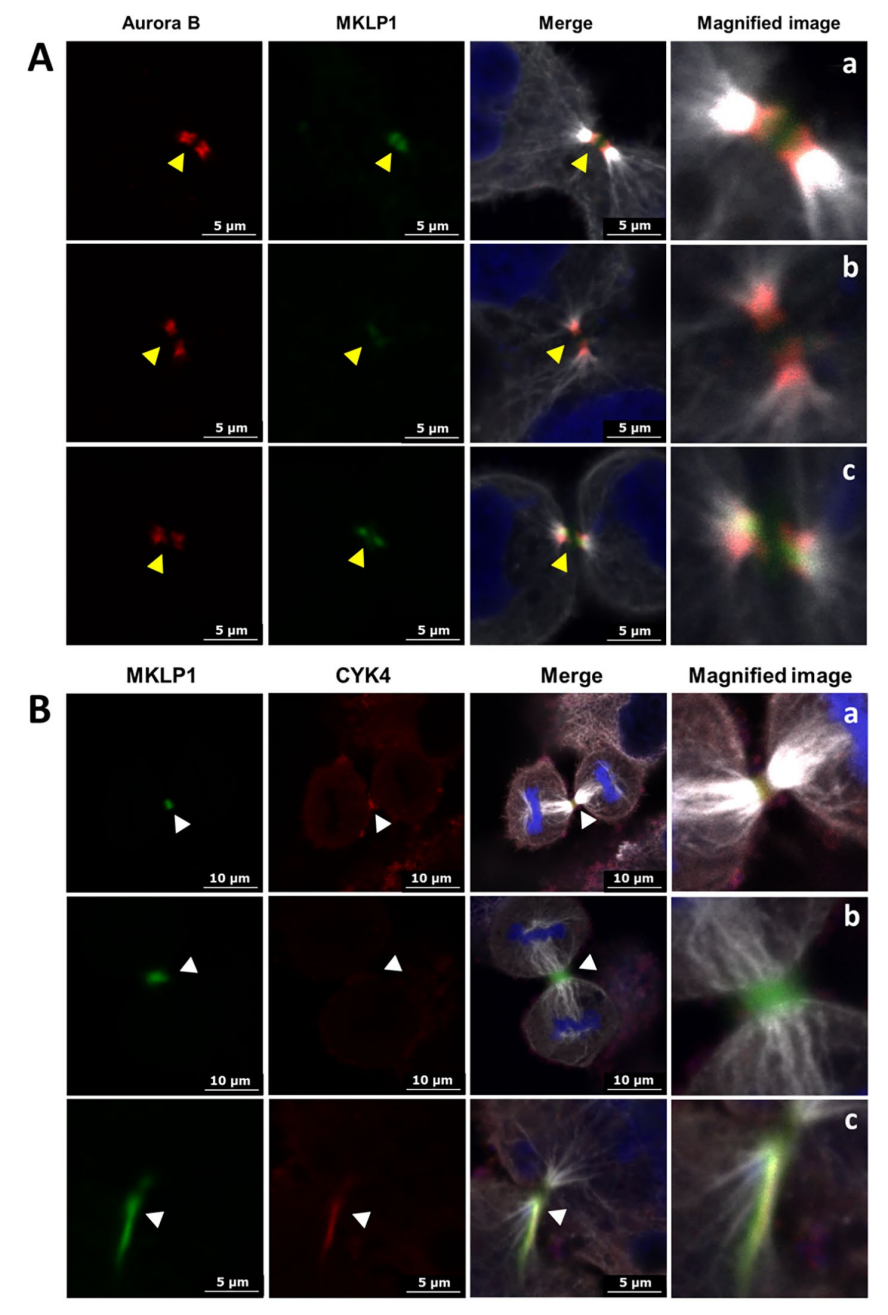
Effects of aSiNPs (20 µg/ml) on the localizations of Aurora B, MKLP1, and CYK4 on midbody during cytokinesis. (**A**) Abnormal co-localization of Aurora B and MKLP1 on midbody (white: microtubule, red: Aurora B, green: MKLP1), (**a**) normal co-localization of Aurora B and MKLP1 on midbody in control group, (**b**) lack of MKLP1 on midbody in aSiNPs treated group, (**c**) incorrect localization of Aurora B and MKLP1 on midbody in aSiNPs treated group. Yellow arrow heads: Aurora B and MKLP1 on midbody. (**B**) Abnormal co-localization of MKLP1 and CYK4 on midbody (white: microtubule, green: MKLP1, red: CYK4), (**a**) normal co-localization of MKLP1 and CYK4 on midbody in control group, (**b**) lack of CYK4 on midbody in aSiNPs treated group, (**c**) incorrect localization of MKLP1 and CYK4 on midbody in aSiNPs treated group. White arrow heads: MKLP1 and CYK4 on midbody

In anaphase and telophase of mitosis, Aurora B translocated to the antiparallel microtubule region of the central spindle. And the S708 site phosphorylated by Aurora B was required for MKLP1 to localize to the central spindle and cluster with CYK4 [24]. Hence, the co-localization of Aurora B and MKLP1 on the midbody was detected. Figure 5A confirmed that the normal co-localization of Aurora B and MKLP1 was altered by aSiNPs. In the control group, MKLP1 and phospho-MKLP1 concentrated on the midbody and colocalized extensively with Aurora B (Fig. 5Aa), while the phenomenon of MKLP1 disappearance (Fig. 5Ab) and incorrect location of Aurora B and MKLP1 on the midbody was spotted in the aSiNPs treated group (Fig. 5Ac).

Furthermore, the correct clustering of centralspindlin complex subunits, MKLP1 and CYK4, on antiparallel microtubule and midbody was essential to the subsequent central spindle bundling and contractile ring formation [16, 17]. Then the co-localization of CYK4 and MKLP1 during cytokinesis was examined. As shown in Fig. 5B, in the control group CYK4 and MKLP1 co-localized with each other very well on the midbody (Fig. 5Ba), while in the aSiNPs treated group lack of CYK4 (Fig. 5Bb) and error position of CYK4 and MKLP1 on the midbody (Fig. 5Bc) were found. Above all, aSiNPs not only suppressed the protein contents of key cytokinesis regulators, but also disrupted the localization of Aurora B and centralspindlin complex on the midbody in L-02 cells.

### PI3K activator IGF reduced the impaction of aSiNPs on aurora B phosphorylation and centralspindlin cluster

**Fig. 6:**
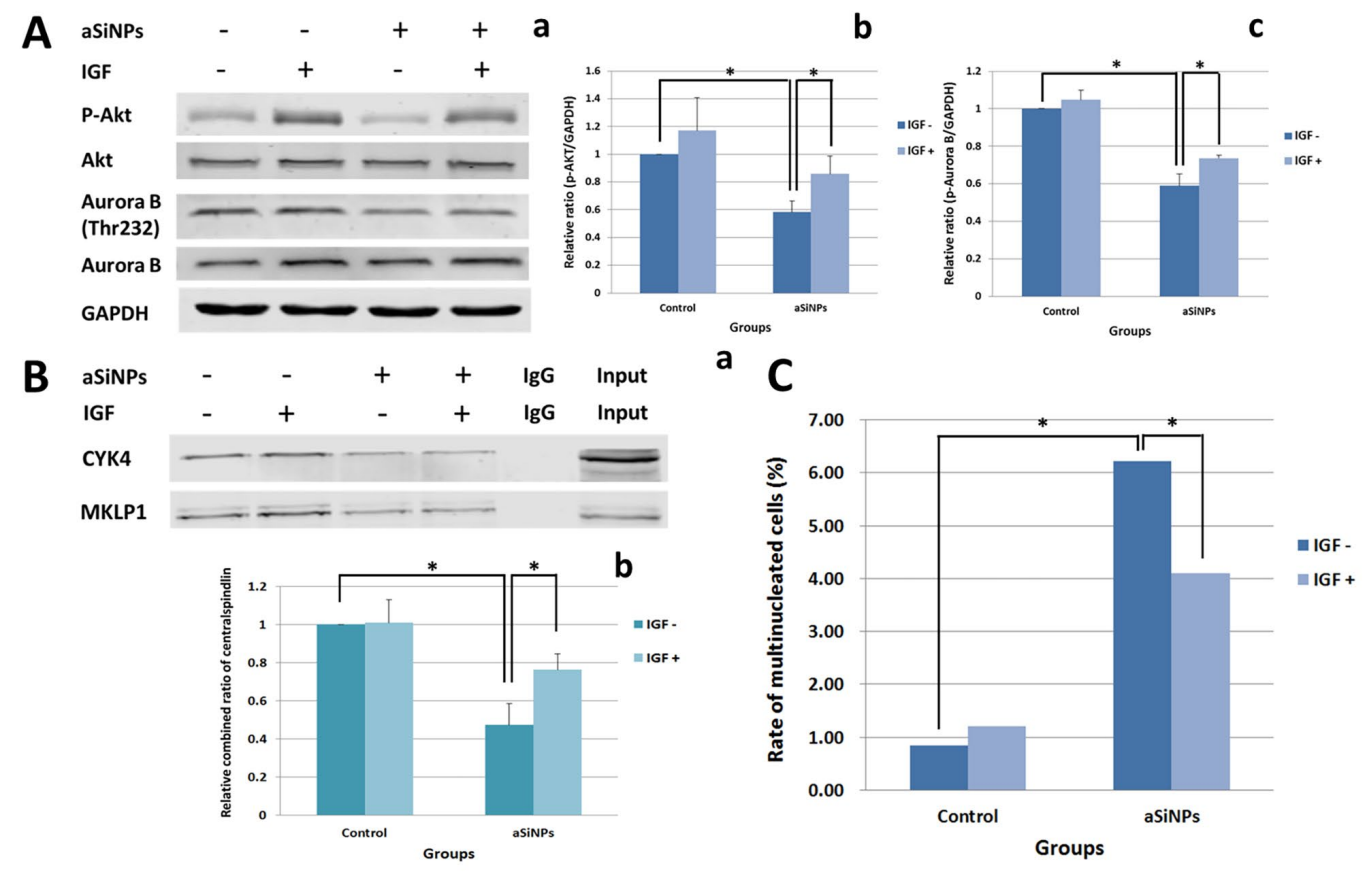
PI3K/Akt pathway activator IGF attenuated the inhibition of Aurora B phosphorylation and centralspindlin cluster induced by aSiNPs. (**A**) Protein content of p-Akt and p-Aurora B was detected by western blot (**a**), and results of relative densitometric analysis were presented in (**b**) and (**c**), respectively. (**B**) Relative ratio of CYK4 bound to MKLP1 was detected by co-immunoprecipitaion to reflect the formation of centralspindlin cluster. Data in (**A**) and (**B**) were expressed as means ± SD from three independent experiments. * *p* < 0.05 compared with control group using one-way ANOVA. (**C**) Rate of multinucleated cells was calculated and data are expressed as frequency. * *p* < 0.05 compared with control group using chi-square test

PI3K activator IGF was introduced in order to confirm whether the influence of aSiNPs on centralspindlin and subsequent cytokinesis was through the inhibition of PI3K/Aurora B signaling. As indicated by pre-experiment, 2 ng/ml IGF pretreated L-02 cells for 1 h could activate PI3K obviously. Thus, the experimental groups were setted as: control group, 2 ng/ml IGF pretreated group, 20 µg/ml aSiNPs treated group, and 20 µg/ml aSiNPs plus 2 ng/ml IGF pretreated group. The result of western blot in Fig. 6A and B manifested that the expression of P-AKT in IGF pretreated group was higher than that of control group, indicating that 2 ng/ml IGF could activate PI3K effectively. ASiNPs could significantly reduce the expression of P-AKT and P-Aurora B. However, compared with the aSiNPs treated group, P-AKT and P-Aurora B expression in aSiNPs plus IGF group had an upward trend. The results showed that the 2 ng/ml IGF could activate the PI3K/Akt pathway and alleviate the decrease of Aurora B phosphorylation caused by aSiNPs.

Co-immunoprecipitation of MKLP1 and CYK4 was further conducted to examine the relative ratio of CYK4 bound to MKLP1, which could reflect the level of centralspindlin complex formation. As exhibited in Fig. 6C, compared with the control group, the relative ratio of CYK4 bound to MKLP1 dramatically reduced in aSiNPs group. However, the relative ratio of colocalization in aSiNPs plus IGF group was 1.5 times as much as the aSiNPs group. Thus, aSiNPs depressed the relative ratio of CYK4 bound to MKLP1 significantly, but this phenomenon was mitigated by the pretreatment of IGF. Meanwhile, the increased rate of multinucleated cells induced by aSiNPs was also reduced owing to IGF (Fig. 6C). Thus, the results of this part suggested that aSiNPs mainly inhibited Aurora B activity through PI3K signaling, thereby affecting the regulation of centralspindlin clustering, resulting in the centralspindlin complex dysfunction and multinucleated cells formation.

### ROS inhibitor NAC reduced the multinucleation effect of aSiNPs

**Fig. 7:**
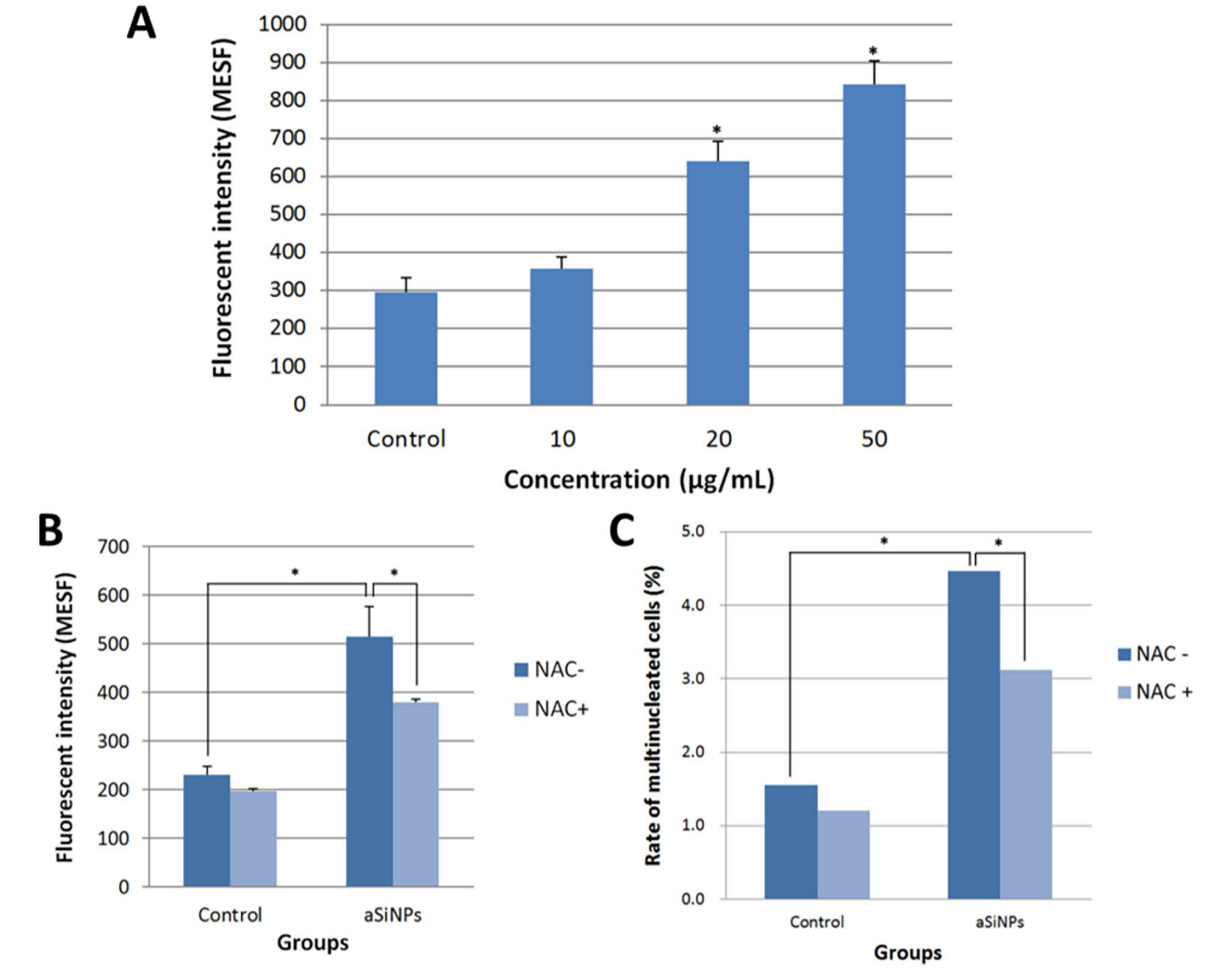
Excessive generation of intracellular ROS in L-02 cells induced by aSiNPs and its relationship with the increase of multinucleation rate. (**A**) Intracellular ROS level was detected after L-02 cells exposed to different concentrations of aSiNPs using flow cytometry, and the corresponding bar graph was shown. ASiNPs increased the intracellular ROS level in a dose-dependent way. (**B**) ROS inhibitor NAC effectively reduced the excessive intracellular ROS induced by aSiNPs. Data in (**A**) and (**B**) were expressed as means ± SD from three independent experiments. * *p* < 0.05 compared with control group using one-way ANOVA. (**C**) ROS inhibitor NAC significantly decreased the rate of multinucleated cells caused by aSiNPs. Rate of multinucleated cells was calculated and data are expressed as frequency. * *p* < 0.05 compared with control group using chi-square test

Oxidative stress has been identified as an important toxic mode of aSiNPs. After exposing L-02 cells to different concentrations of aSiNPs for 24 h, the intracellular ROS level was detected. Figure 7 A showed the aSiNPs induced excessive generation of ROS as aSiNPs concentration increasing. Compared with the control, intracellular ROS level in 20 and 50 µg/ml aSiNPs treated group was significantly increased, which raised around to twice and treble, respectively.

To further explore the relationship between excessive intracellular ROS and the multinucleation effect of aSiNPs, ROS inhibitor NAC was used to suppress the aSiNPs-induced oxidative damage. The result of pre-experiment confirmed that 5 mM NAC pretreated L-02 cells for 2 h could effectively inhibit the excessive generation of ROS. Thus, the experimental groups were setted as: control group, 5 mM NAC pretreated group, 20 µg/ml aSiNPs treated group, and 20 µg/ml aSiNPs plus 5 mM NAC pretreated group. As shown in Fig. 7B, ROS level in aSiNPs plus NAC group significantly decreased compared with the aSiNPs treated group. And in Fig. 7C, the rate of multinucleated cells in aSiNPs plus NAC group showed a significant drop from 4.5 to 3.1%, compared with the aSiNPs group. The results suggested that 5 mM NAC could effectively suppressed intracellular ROS level and multinucleated cells caused by aSiNPs. Thus, aSiNPs might lead to multinucleation through excessive ROS and oxidative damage.

### NAC reduced the impaction of aSiNPs on PI3K 110β/aurora B pathway and cytokinesis regulatory proteins

**Fig. 8:**
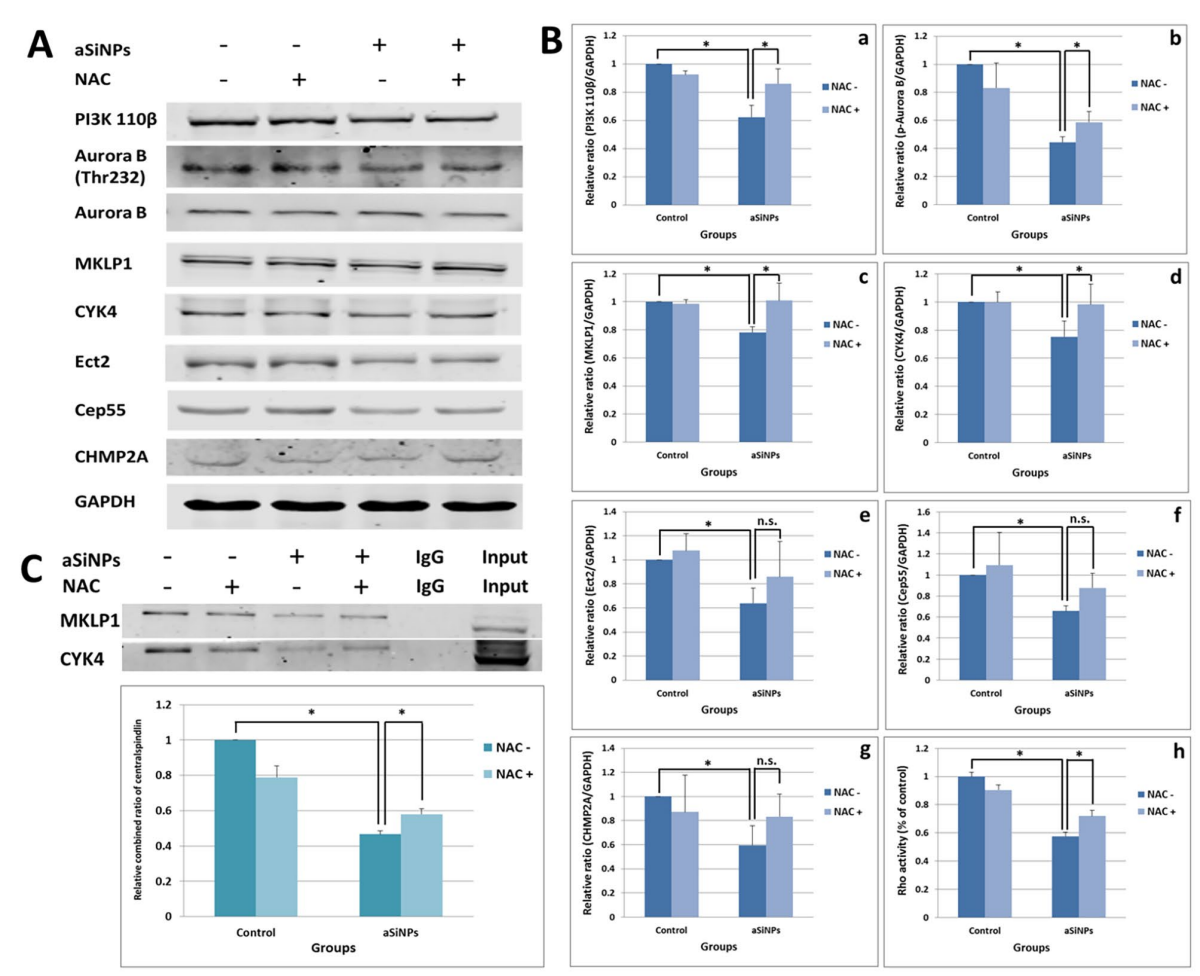
ROS inhibitor NAC attenuated the inhibition of PI3K 110β/Aurora B pathway and cytokinesis related regulation proteins induced by aSiNPs. (**A**) Results of western blot analysis. (**B**) Relative densitometric analysis of the protein bands (**a**-**g**) and detection of Rho activity (**h**) were performed and presented. (**a**) PI3K 110β, (**b**) p-Aurora B, (**c**) MKLP1, (**d**) CYK4, (**e**) Ect2, (**f**) Cep55, (**g**) CHMP2A, (**h**) Rho activity detected by G-LISA biochem kit. (**C**) Relative ratio of CYK4 bound to MKLP1 was detected by co-immunoprecipitaion to reflect the formation of centralspindlin cluster. Data were expressed as means ± SD from three independent experiments. * *p* < 0.05 compared with control group using one-way ANOVA.

To further investigate the specific mechanism by which aSiNPs induced abnormal cytokinesis via oxidative stress, the influence of NAC on the down-regulation of PI3K/Aurora B signaling and related cytokinesis regulatory proteins induced by aSiNPs was investigated. The experimental groups were setted as: control group, 5 mM NAC pretreated group, 20 µg/ml aSiNPs treated group, and 20 µg/ml aSiNPs plus 5 mM NAC pretreated group. As revealed in Fig. 8A and B, the expression of PI3K 110β and the phosphorylation of Aurora B were decreased by aSiNPs. However, there was a rise trend in aSiNPs plus NAC group, compared with the aSiNPs group. Similarly, the down regulation of the protein contents of cytokinesis regulators, including MKLP1, CYK4, Ect2, Cep55, and CHMP2A, as well as Rho activity, resulting from aSiNPs were attenuated by NAC involvement. Especially, the level of MKLP1 and CYK4 in aSiNPs plus NAC group was around 1.25 times as high as the aSiNPs group. Meanwhile, the effect of NAC on aSiNPs induced abnormal cluster of centralspindlin was assessed by co-immunoprecipitation assay. The results in Fig. 8C manifested that aSiNPs inhibited the combination of MKLP1 and CYK4, and NAC improved CYK4 bound to MKLP1 obviously in the aSiNPs treated group. The relative ratio of colocalization in aSiNPs plus NAC group was higher than the aSiNPs group. This obtained data indicated that excessive ROS and oxidative damage were involved in aSiNPs-induced down regulation of the PI3K 110β/Aurora B pathway as well as dysfunction of the centralspindlin complex.

## Discussion

With the widespread use of aSiNPs, the risk of people’s exposure to aSiNPs is also increasing, which causes great harm to the health of human beings [7]. Respiratory inhalation was the primary way that aSiNPs enter the body, and then transportation of aSiNPs from the lungs into the circulatory system was possible [28, 29]. Phagocytosis of hepatic Kupffer cells might result in the entry of aSiNPs from the blood into the liver and promote their internalization by hepatocytes. At the same time, studies have shown that the liver was one of the important target organs of aSiNPs. It was reported that aSiNPs deposited in the liver and caused severe damage, such as vacuolar degeneration of hepatocytes and focal necrosis [30, 31]. In order to simulate the exposure mode that aSiNPs entered the human body through respiratory inhalation, we established a mice model of transtracheal instillation. The pathological analyses of the liver and lung tissue showed that aSiNPs induced nuclear fragmentation, vacuolization, and necrosis of hepatocytes near the central vein in the liver tissue and lymphocyte infiltration, pulmonary interstitial thickening and bronchial epithelial damage in lung tissue (Fig. 1A and B).

In addition to the pathological damage observed in the aSiNPs treated group, the significantly increased proportion of multinucleated cells was more worthy of our attention. Normally, the liver tissue showed a certain degree of multinucleated cells under healthy conditions, which might be related to the higher metabolic level. However, our study showed that aSiNPs induced a significant increase in the multinucleation rate of hepatocytes, and the number of multinucleated bronchial epithelial cells was also increased (Fig. 1A and B). Unscheduled multinucleated cells, on the one hand, underwent apoptotic or mitotic catastrophe, which would cause further tissue damage, on the other hand, underwent malignant transformation owing to chromosomal instability [12, 13]. It was reported, multinucleation was one of the manifestations of polyploidization, and polyploidy, a feature of many human cancers, might predispose to genomic instability and aneuploidization which played a major role in carcinogenesis [32]. Meanwhile, our study has demonstrated that aSiNPs could induce multinucleation of L-02 cells in vitro as well. Moreover, the multinucleation effect of smaller aSiNPs (46 nm) was significantly stronger than that of larger aSiNPs (64 nm) (Fig. 1C). This might be related to the fact that the toxicity of aSiNPs was influenced by its size. Thus, 46 nm aSiNPs was used for subsequent in vitro studies to explore the underlying mechanisms of how aSiNPs-induce increased abnormal multinucleated cells.

Our preliminary investigation found that the contraction ring could not be constricted effectively, resulting in the rebound of the constriction and then the direct fusion of the two daughter cells after aSiNPs treatment [9]. Additionally, the phenomenon that L-02 cells exposed to aSiNPs could not separate the two daughter cells effectively, instead leaving a thin and long intercellular bridge between each other were also observed [9]. Similarly, this result was also obtained in this study. ((Fig. 3B) Therefore, cytokinesis failure was the main reason for aSiNPs-induced multinucleation. Other research showed that cytokinesis failure induced by actin cytoskeleton disassembly was one of the major mechanisms of cellular ploidy in hepatocytes [33]. The actin cytoskeleton consisted of microfilaments and their accessory and regulatory proteins, which were involved in the assembly of contractile rings during cytokinesis [34, 35]. Our research indicated that microfilaments, as the main body of the contractile ring, were abnormal in the structure after aSiNPs treatment, mainly manifested as seen in microfilaments agglomeration and disordered arrangement (Fig. 2B and C). A clear co-localization between aSiNPs and aggregated microfilaments was also observed (Fig. 2C). This suggested that aSiNPs could enter the cytoplasm and directly affect the microfilament structure. Microfilaments agglomeration was detrimental to contractile ring assembly during cytokinesis. Microtubules were another member of the cytoskeleton that controlled the segregation of chromosomes, the placement of contractile ring and the completion of cell cleavage during mitosis [36]. In this study, the morphology and structure of microtubules did not change obviously after aSiNPs treatment, but the distribution of microtubules in the perinuclear cytoplasmic region was affected by the agglomerated microfilaments (Fig. 3A). Furthermore, micronuclei was observed in the aSiNPs treated group (Fig. 3B). The reason for this is that, during mitosis, the nuclear membrane disappeared, and then aSiNPs got the opportunity to directly interact with the chromosomes and caused micronuclei formation. Taken together, aSiNPs entering the cells could directly induce chromosome breakage and cytoskeletal rearrangements, mainly including microfilaments aggregation and altered perinuclear distribution of microtubules. Damage to the cytoskeletal structure by aSiNPs could be the trigger for the failure of cytokinesis. However, the mechanism of cytokinesis failure induced by aSiNPs needs further discussion.

Contractile ring assembly, cleavage furrow ingression and abscission were important links in cytokinesis [35]. Centralspindlin was a key microtubule organizer and signaling hub for cytokinesis. It was composed of two proteins: a kinesin-like protein, Mitotic kinesin-like protein 1 (MKLP1), and a Rho GTPase activating protein (RhoGAP), CYK-4 [16]. Centralspindlin organized antiparallel arrays of microtubule at the spindle midzone and midbody, and recruited cytokinetic effector proteins to promote cytokinesis [37]. CYK4 as one of the subunits of centralspindlin together with downstream cytokinetic effector proteins, including Ect2 and small GTPase RhoA, were the guarantee of contractile ring assembly and cleavage furrow ingression. CYK4 recruited Ect2 to the centralspindlin complex, and then Ect2 drove local activation of the small GTPase RhoA, which controls actomyosin contractility and subsequent groove entry [38]. In our study, aSiNPs could reduce the expression of CYK4, Ect2 and the activity of RhoA in L-02 cells (Fig. 4A C). Microfilaments agglomeration, coupled with abnormal expression of CYK4 and downstream cytokinetic effector proteins were one of the testimonies that aSiNPs could lead to abnormal function of contractile rings and cleavage furrow ingression during cytokinesis. MKLP1 as a kinesin-like motor protein could move along microtubule to the spindle midzone, finally concentrating at the midbody and playing an important role in abscission during cytokinesis [37]. Cep55 and CHMP2A were the downstream cytokinetic effector proteins of MKLP1, which acted a leading role in the process of abscission [39, 40]. Abscission depended on the endosomal sorting complex required for transport (ESCRT) machinery, and a sub-complex of this machinery ESCRT-III was the main driver of membrane remodeling processes [41]. CHMP2B was a relatively recent acquisition in the evolution of the ESCRT-III complex, and it acted in most ESCRT-catalyzed membrane remodeling processes [42]. The assembly of the ESCRT machinery at the midbody was initiated by Cep55 [43], where Cep55 positioning at the midbody depended on the centralspindlin subunit MKLP1 [44]. In addition to membrane remodeling, maturation of the spindle midzone into a stable midbody was a prerequisite for abscission. Cep55 was required for the establishment and proper function of the midbody structure. It has been shown that in Cep55 knockdown cells, structural and regulatory components of the midbody were either absent or mislocalized [44]. Cep55 and CHMP2A, whose expressions were decreased after exposed aSiNPs in our study (Fig. 4A and B). Therefore, the reason that aSiNPs hindered abscission might be due to the maturation of the spindle midzone and membrane remodeling. In summary, aSiNPs caused cytokinesis failure by downregulating the protein expression of two subunits of the centralspindlin and downstream cytokinetic effector proteins.

Meanwhile, at anaphase onset, centralspindlin precisely localized to the plus ends of the antiparallel microtubule where it regulated and ensured the progression of normal cytokinesis. Studies have shown that the positioning of centralspindlin depends on the catalytic activity of MKLP1 [45]. Aurora B was the enzymatic heart of the chromosomal passenger protein complex (CPC) that regulated key cytokinesis events, such as activation of the central spindle assembly and construction and regulation of the contractile apparatus [46]. It was reported that Aurora B phosphorylated MKLP1 at the highly conserved site S708, which further regulated the clustering and localization of centralspindlin [25, 47]. Nicolas Taulet et al. demonstrated that the disfunction of Aurora B was related to the decrease of phospho S708 MKLP1 observed at the central spindle [48]. As shown in our study, the phosphorylation level of Aurora B was down regulated by aSiNPs significantly. (Figure 4A and B), and the normal co-localization of Aurora B and MKLP1 on the midbody was also altered (Fig. 5A), which indicated that aSiNPs decreased the phosphorylation level of Aurora B, and then MKLP1 could not be effectively activated. Meanwhile, decreased catalytic activity of MKLP1 could adversely affect the positioning of centralspindlin at the plus ends of antiparallel microtubule. As known, CYK4 binding promoted antiparallel bundling of microtubules by MKLP1 and accumulation of centralspindlin into the antiparallel microtubule overlap. MKLP1 and CYK4 subunits are essential for microtubules bundling, and neither MKLP1 alone nor CYK4 alone can efficiently bundle microtubules [18]. The normal binding of CYK-4 and MKLP1 was an important prerequisite for the binding of microtubules by centralspindlin. In our study, the co-localization of CYK4 and MKLP1 was abnormal during cytokinesis, mainly manifested by the lack of CYK-4 and the mislocalization of CYK4 and MKLP1 on the intermediate in the aSiNPs-treated group (Fig. 5B). In short, decreased level of Aurora B phosphorylation induced by aSiNPs was an obstacle to centralspindlin clustering and correct positioning. Afterwards, we further explored the reasons for the decreased phosphorylation level of Aurora B in L-02 cells treated with aSiNPs.

The PI3K/Akt signaling pathway was closely related to the proliferation and differentiation of cells [49]. From our previous transcriptomic analyses, it was found that aSiNPs could cause a significant down-regulation of the PI3K/Akt signaling pathway. Other studies have shown that the PI3K pathway regulated cytoskeletal dynamics in an Akt-independent manner [50]. After PI3K activation by receptors, its class IA isoforms (p110α and p110β) generated lipid second messengers, which initiated multiple signal transduction cascades, where PI3K 110β possessed kinase-independent functions in regulating cell proliferation [50]. At the same time, studies have also reported that PI3K 110β could affect the protein activity of Aurora B, and subsequently regulated cytokinesis through Aurora B [22]. In this study, the expression of PI3K, especially the p110β subunit, was significantly reduced after L-02 cells were treated with aSiNPs. Afterwards, we used the PI3K activator IGF to reversely verify the regulatory relationship between PI3K and Aurora B. Firstly, IGF could significantly enhance the phosphorylation level of Akt, a downstream molecule of PI3K, which indicated that IGF could effectively activate the PI3K. The results also suggested that compared with the aSiNPs -treated group, IGF induced a higher level of binding of CYK4 to MKLP1 (Fig. 6B) by reversing the phosphorylation level of Aurora B at the Thr232 site (Fig. 6A), which ultimately reduced the rate of multinucleated cells (Fig. 6C). Therefore, it could be considered that aSiNPs might inhibit the PI3K/Aurora B signaling pathway, which affected centralspindlin positioning and clustering, consequently inducing multinucleated cell formation.

As previously mentioned, aSiNPs induced ROS-dependent oxidative stress, which was one of the recognized toxic modes of aSiNPs. Excessively increased ROS might cause damage to biological macromolecules, including DNA, proteins, etc. [51, 52]. Our research group has previously confirmed that the excessive production of ROS induced by aSiNPs was related to the multinucleation of cells [9]. On the contrary, the phenomenon that using NAC to inhibit the level of ROS could significantly reduce the multinucleation rate was observed in our present study (Fig. 7A C). Meanwhile, after NAC treatment, the protein expression of the PI3K/ Aurora B signaling pathway gradually recovered, and then the centralspindlin complex subunits and its downstream cytokinetic effector proteins, including Ect2, Cep55, CHMP2A and RhoA, also showed the same trend compared with the aSiNPs treated group (Fig. 8A and B). Meanwhile, NAC also improved CYK4 and MKLP1 binding levels (Fig. 8C). Therefore, excessive ROS could not only directly regulate the expression of centralspindlin and subsequent cytokinesis, but also could affect the clustering of the centralspindlin complex subunits, through the PI3K / Aurora B signaling pathway.

## Conclusion

**Fig. 9:**
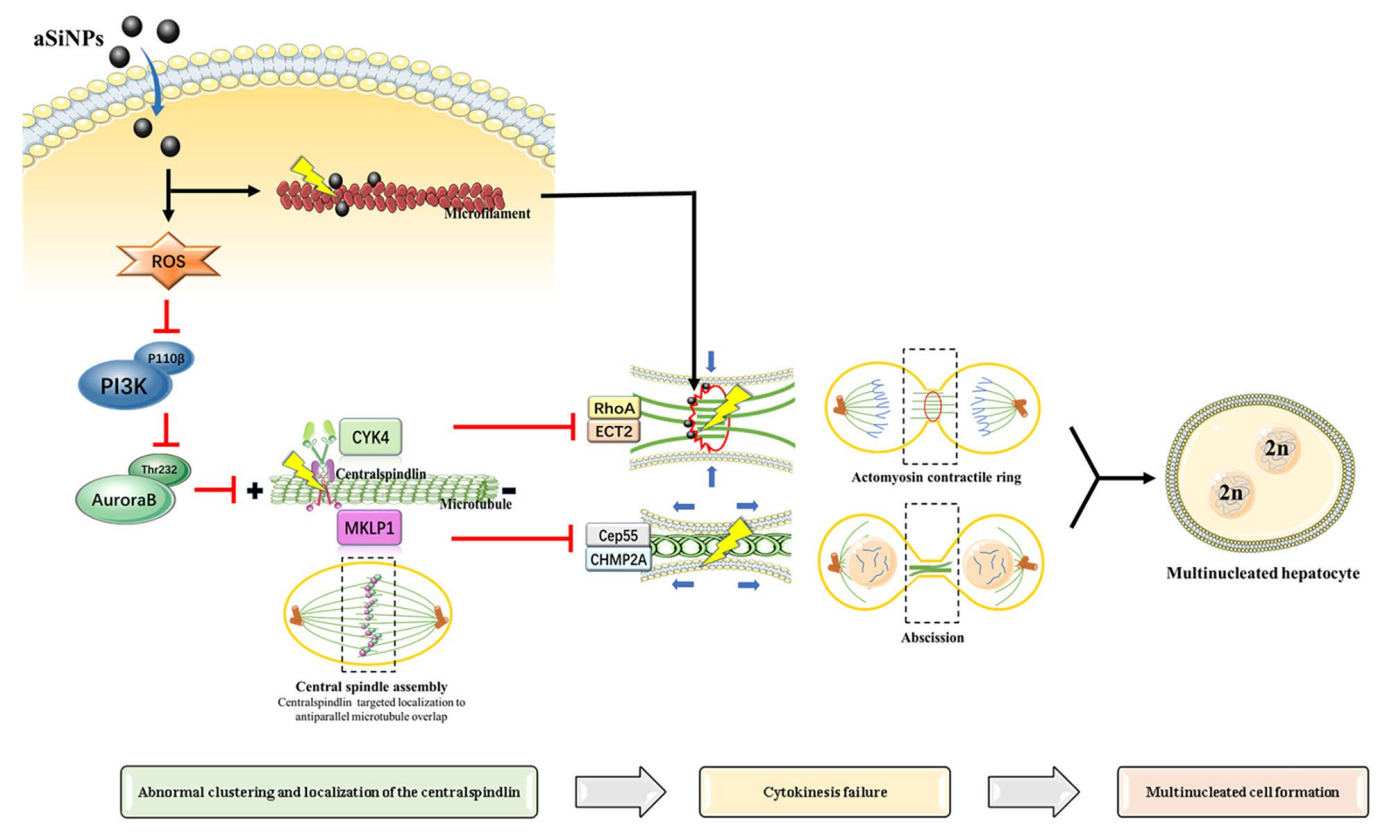
A schematic of the molecular mechanisms shown that aSiNPs-induced abnormal cytokinesis and multinucleation

As shown in Fig. 9, after aSiNPs entered the cell, on the one hand, it directly damaged the cytoskeleton and chromosome through mechanical action or surface activity, and on the other hand, it induced ROS-dependent oxidative stress. Excessive ROS could not only reduce the contents or expression of cytokinesis related proteins, but also led to abnormal centralspindlin location and function through the PI3K/Aurora B signaling pathway, resulting in abnormal function of contractile rings and incomplete abscission. Taken together, aSiNPs caused abnormal morphology and function of microfilaments, while also having affected the function of centralspindlin as well as cytokines related proteins; which ultimately resulted in cytokinesis failure and the formation of multinucleated cells.

### Electronic supplementary material

Below is the link to the electronic supplementary material.

**Supplementary Figure 1**. Morphology and particle size of aSiNPs. (A) TEM images. (B) Average size and size distribution. Both two sizes aSiNPs possessed spherical or ellipsoidal shape and the average sizes measured by Image J software were 64 and 46 nm, respectively.

**Supplementary Table 1**. Hydrodynamic size and Zeta potential of two silicon nanoparticles in dispersion media.

## Data Availability

The datasets during and /or analyzed during the current study are available from the corresponding author upon reasonable request.
