# Supplementary material for: Amorphous silica nanoparticles cause abnormal cytokinesis and multinucleation through dysfunction of the centralspindlin complex and microfilaments
Source: Part Fibre Toxicol. 2023 Aug 22;20:34. doi: 10.1186/s12989-023-00544-8 (PMC10464468; doi:10.1186/s12989-023-00544-8)
Supplement: Supplementary file 1 — Supplementary Figure 1. Morphology and particle size of aSiNPs. (A) TEM images. (B) Average size and size distribution. Both two sizes aSiNPs possessed spherical or ellipsoidal shape and the average sizes measured by Image J software were 64 and 46 nm, respectively. [file 12989_2023_544_MOESM1_ESM.docx]

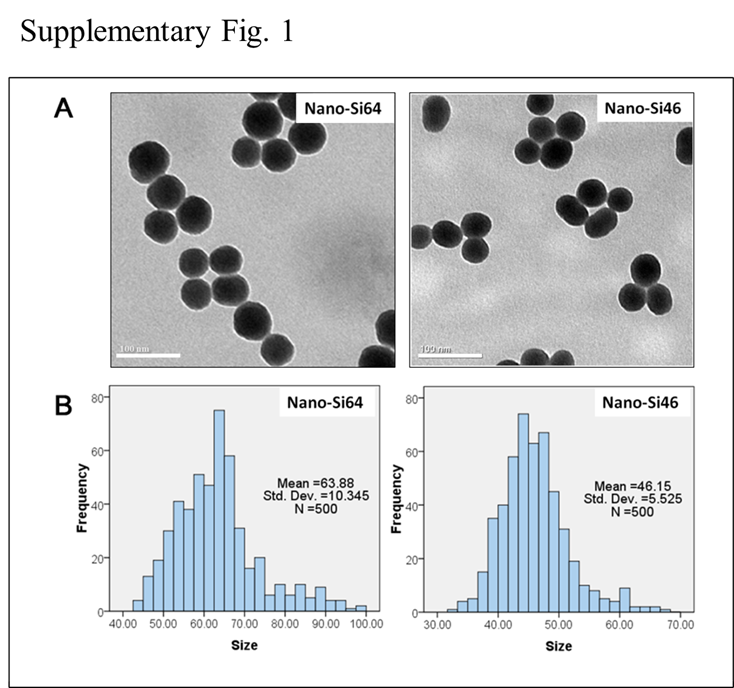


**Supplementary Figure 1.** Morphology and particle size of aSiNPs. (A) TEM images. (B) Average size and size distribution. Both two sizes aSiNPs possessed spherical or ellipsoidal shape and the average sizes measured by Image J software were 64 and 46 nm, respectively.
