# Supplementary material for: Amorphous silica nanoparticles cause abnormal cytokinesis and multinucleation through dysfunction of the centralspindlin complex and microfilaments
Source: Part Fibre Toxicol. 2023 Aug 22;20:34. doi: 10.1186/s12989-023-00544-8 (PMC10464468; doi:10.1186/s12989-023-00544-8)
Supplement: Supplementary file 2 — Supplementary Table 1. Hydrodynamic size and Zeta potential of two silicon nanoparticles in dispersion media. [file 12989_2023_544_MOESM2_ESM.doc]

Supplementary Table 1. Hydrodynamic size and Zeta potential of two silicon nanoparticles in dispersion media.

| Silicon nanoparticles | Time (h) | Distilled water | | |  | 1640 cell culture medium | | |
| --- | --- | --- | --- | --- | --- | --- | --- | --- |
|  |  | Size (nm) | PDI | Zeta potential (mV) |  | Size (nm) | PDI | Zeta potential (mV) |
| Nano-Si64 | 0 | 108.32 | 0.113 | - 32.0 |  | 107.53 | 0.114 | - 29.8 |
|  | 3 | 110.02 | 0.086 | - 33.2 |  | 106.92 | 0.105 | - 31.8 |
|  | 6 | 109.06 | 0.113 | - 32.7 |  | 108.11 | 0.097 | - 31.5 |
|  | 12 | 109.77 | 0.083 | - 31.6 |  | 110.07 | 0.122 | - 31.5 |
|  | 24 | 109.10 | 0.087 | - 32.4 |  | 108.04 | 0.127 | - 28.4 |
| Nano-Si46 | 0 | 68.14 | 0.095 | - 40.6 |  | 70.08 | 0.116 | - 27.8 |
|  | 3 | 66.50 | 0.084 | - 38.4 |  | 68.86 | 0.075 | - 30.1 |
|  | 6 | 65.68 | 0.049 | - 37.6 |  | 67.18 | 0.062 | - 30.0 |
|  | 12 | 67.30 | 0.080 | - 36.3 |  | 67.82 | 0.100 | - 29.9 |
|  | 24 | 66.09 | 0.049 | - 38.2 |  | 68.35 | 0.089 | - 29.5 |

| Silicon nanoparticles | Time | Physiological saline | | |
| --- | --- | --- | --- | --- |
|  |  | Size (nm) | PDI | Zeta potential (mV) |
| Nano-Si64 | 10 min | 110.4 | 0.119 | - 39.2 |
|  | 1 h | 111.8 | 0.127 | - 41.3 |
|  | 6 h | 108.1 | 0.142 | - 40.7 |
|  | 12 h | 108.8 | 0.141 | - 38.3 |
|  | 24 h | 107.8 | 0.136 | - 43.8 |
